# Functional paralysis of GM-CSF–derived bone marrow cells productively infected with ectromelia virus

**DOI:** 10.1371/journal.pone.0179166

**Published:** 2017-06-12

**Authors:** Lidia Szulc-Dąbrowska, Justyna Struzik, Agnieszka Ostrowska, Maciej Guzera, Felix N. Toka, Magdalena Bossowska-Nowicka, Małgorzata M. Gieryńska, Anna Winnicka, Zuzanna Nowak, Marek G. Niemiałtowski

**Affiliations:** 1 Department of Preclinical Sciences, Faculty of Veterinary Medicine, Warsaw University of Life Sciences-SGGW, Warsaw, Poland; 2 Analytical Center, Warsaw University of Life Sciences-SGGW, Warsaw, Poland; 3 Department of Veterinary Medicine, University of Cambridge, Cambridge, United Kingdom; 4 Department of Biomedical Sciences, Ross University School of Veterinary Medicine, Basseterre, St. Kitts, West Indies; 5 Department of Pathology and Veterinary Diagnostics, Faculty of Veterinary Medicine, Warsaw University of Life Sciences-SGGW, Warsaw, Poland; 6 Department of Genetics and Animal Breeding, Faculty of Animal Sciences, Warsaw University of Life Sciences-SGGW, Warsaw, Poland; CEA, FRANCE

## Abstract

Ectromelia virus (ECTV) is an orthopoxvirus responsible for mousepox, a lethal disease of certain strains of mice that is similar to smallpox in humans, caused by variola virus (VARV). ECTV, similar to VARV, exhibits a narrow host range and has co-evolved with its natural host. Consequently, ECTV employs sophisticated and host-specific strategies to control the immune cells that are important for induction of antiviral immune response. In the present study we investigated the influence of ECTV infection on immune functions of murine GM-CSF–derived bone marrow cells (GM-BM), comprised of conventional dendritic cells (cDCs) and macrophages. Our results showed for the first time that ECTV is able to replicate productively in GM-BM and severely impaired their innate and adaptive immune functions. Infected GM-BM exhibited dramatic changes in morphology and increased apoptosis during the late stages of infection. Moreover, GM-BM cells were unable to uptake and process antigen, reach full maturity and mount a proinflammatory response. Inhibition of cytokine/chemokine response may result from the alteration of nuclear translocation of NF-κB, IRF3 and IRF7 transcription factors and down-regulation of many genes involved in TLR, RLR, NLR and type I IFN signaling pathways. Consequently, GM-BM show inability to stimulate proliferation of purified allogeneic CD4^+^ T cells in a primary mixed leukocyte reaction (MLR). Taken together, our data clearly indicate that ECTV induces immunosuppressive mechanisms in GM-BM leading to their functional paralysis, thus compromising their ability to initiate downstream T-cell activation events.

## Introduction

Ectromelia virus (ECTV) is a member of the *Poxviridae* family, genus *Orthopoxvirus* and is the causative agent of mousepox, a disease called “smallpox of mice”. ECTV is closely related to variola virus (VARV)–the causative agent of smallpox responsible for millions of death in the history of mankind. Another member of orthopoxviruses–monkeypox virus (MPXV), is a zoonotic agent that causes a human disease with high mortality and clinical signs very similar to smallpox. Rimoin et al. [1] reported a dramatic increase in human monkeypox incidence in rural Democratic Republic of Congo. Moreover, the monkeypox outbreak in the United States of America in 2003 demonstrated that MPXV is capable of spreading to new animal reservoirs outside central Africa. In this case prairie dogs were infected by rodents imported from Ghana and served as amplification vectors, ultimately transmitting disease to humans [1]. It is not excluded that the increased frequency of MPXV infection in humans, especially in immunocompromised individuals, may permit MPXV to evolve and maintain itself independently in the human population [2]. Cessation of vaccination against smallpox has created a real threat since VARV and MPXV can be used as potential agents of bioterrorism [3].

Our current understanding of smallpox disease comes from clinical data from humans vaccinated with vaccinia virus (VACV) and from animal studies using VACV and other closely related viruses, such as ECTV, MPXV, cowpox virus (CPXV). In original vaccines against smallpox, CPXV and VACV were used to prevent the onset and spread of the disease, what eventually led to eradication of smallpox from the world. Although this can be classified as one of the most spectacular human achievements in history of vaccinology, the safety of these vaccines requires improvement [4]. Fortunately, the mousepox model is still the most versatile model to study pathogenesis of smallpox and other generalized viral infections, as well as genetic resistance to disease and viral immunobiology. The use of ECTV as a model for smallpox stems from several important common properties of these viruses. Firstly, ECTV, like VARV, but in contrast to VACV and CPXV, has a restricted host replication phenotype and has coevolved with its natural host. Secondly, ECTV and VARV are highly infectious and cause severe, acute systemic disease with high mortality rates in their natural hosts [5]. Further similarities between mousepox and smallpox viruses include: replication strategy and transmission, cytokine responses in host cells, aspects of pathology and development of characteristic pock lesions on the skin during later stages of infection [6].

Conventional dendritic cells (cDCs) are professional antigen presenting cells (APCs) capable of initiating primary T cell-mediated immune responses with high efficiency. They can differentiate from both clonal common lymphoid progenitors (CLPs) and clonal common myeloid progenitors (CMPs), however CLPs are more efficient at producing thymic cDCs, whereas CMPs are more potent at generating splenic and lymph node (LN) cDCs [7]. In non-lymphoid tissues cDCs remain in a state which is defined as immature e.g., Langerhans cells (LCs) within all epithelia [8]. Upon infection with pathogens or administration of microbial products (e.g.,lipopolysaccharide—LPS), or induction of danger signals from tissue damage, cDCs undergo maturation. This processis accompanied by up-regulation of the expression of major histocompatibility complex class II (MHC II) and costimulatory molecules such as CD80, CD86, and CD40, and increased capacity to produce interleukin (IL)-12 and other cytokines by cDCs [9]. Moreover, upon maturation, cDCs rapidly switch their chemokine receptor repertoire, they down-regulate inflammatory chemokine receptors and up-regulate chemokine receptors for secondary lymphoid organ chemokines. Activated cDCs migrate to the T cell-rich region of secondary lymphoid tissues, where they may encounter and stimulate naive T cells [10]. Depending on the lineage, maturation stage and activation signals cDCs are able to induce different types of T helper (Th) cell responses. Interaction between cDCs and T cells through CD40-CD40L ligation leads to optimal cytokine production by DCs, especially IL-12, a Th1-type cytokine [8,11].

The ability of cDCs to elicit polarized Th cell differentiation depends on maturation and activation state of cDCs. Immature cDCs show strong ability to release tumor necrosis factor (TNF)-α whereas mature cDCs have a great ability to release IL-12 in response to pathogen stimuli [12]. It has been reported that individual antigens can stimulate cDCs to induce either Th1 or Th2 immune responses dependent on the production of IL-12, a soluble factor that has been implicated in the development of naive precursors into Th1 cells upon activation with LPS or derived signals such as CD40L co-stimulation [11,13]. Meanwhile, orthopoxviruses, including ECTV, encode a repertoire of proteins that are engaged in modulation of cDC maturation and function [14]. VACV abortively replicates in cDCs, alters maturation of immature cDCs,and consequently inhibits T cell activation [15]. On the contrary, Yao et al. [16] revealed that VACV infection induces dendritic cell maturation but inhibits antigen presentation through MHC class II. Moreover, VACV-infected APCs have been shown to prime naive T cells *in vivo*, probably owing to a rapid endogenous viral antigen presentation, which occurs too fast such that the virus is unable to alter APC activation of CD8^+^ T cells [17]. Undeniably, VACV inhibits the ability of DCs to perform various innate and adaptive functions. This includes: alteration in expression of MHC class II molecules on the cell surface [18,19], inhibition of antigen processing [16,20] and alteration in DC migration [21]. Additionally, Hansen et al. [22] reported an interesting study showing inhibition of human dendritic cell function by CPXV. The nonproductive CPXV infection of cDCs fails to induce cytokine secretion and inhibits stimulation of allogeneic CD4^+^ T cell proliferation.

Due to the high host-restriction of ECTV (the mouse is the only known natural host of the virus [23]), the viral strategies to control murine cDCs may be more sophisticated and extensive than those observed in VACV or CPXV infected human DCs. Indeed, our results show for the first time that ECTV productively infects granulocyte-macrophage colony stimulating factor (GM-CSF)–cultured bone marrow (GM-BM) cells, comprised of cDCs and macrophages, and leads to their functional paralysis. Infected GM-BM are unable to uptake and process antigen, display inability to mature and show dysfunction in mounting proinflammatory and T cell responses. Decreased cytokine/chemokineproduction may result from impaired ligand-induced accumulation of nuclear factor (NF)-κB, interferon-regulatory factor (IRF)3 and IRF7 within the nucleus and down-regulation of many genes involved in Toll-like receptor (TLR), retinoic acid-inducible gene-I (RIG-I)-like receptor (RLR), nucleotide-binding oligomerization domain (NOD)-like receptor (NLR) and interferon (IFN) type I signaling pathways. Taken together, our results demonstrate that a single virus may affect GM-BM functions at multiple levels of their physiology leading to complete inability to fulfill their innate and acquired immune functions.

## Results

### ECTV productively infects GM-BM, including cDCs, and is released from the infected cells

Since other orthopoxviruses, such as VACV [24] and CPXV [22], have been shown to fail their replication in human cDCs, we asked whether ECTV is able to productively infect cDCs derived from its natural host–the mouse. We used GM-BM, which have been described as a heterogeneous population comprised of cDCs and macrophages [25]. GM-BM were additionally purified by MACS positive selection using anti-CD11c microbeads [26] to obtain a cell population enriched in cDCs. As shown in S1 Fig after MACS separation the population of CD11c^high^ cells contained a high percentage of MHC II^+^ cells and exhibited high expression of CD205 molecules.

**Fig 1:**
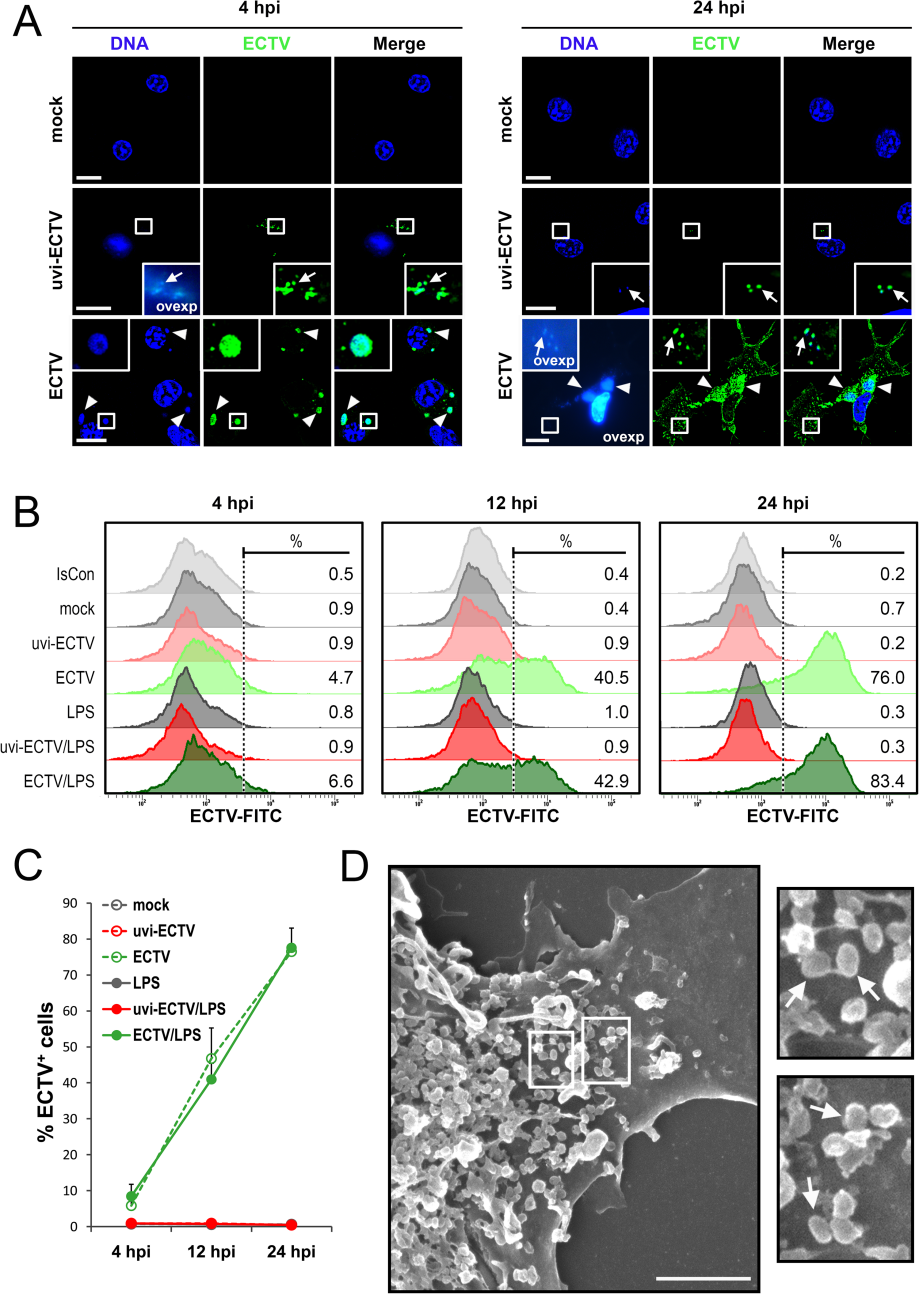
Kinetics of ECTV replication in GM-BM. GM-BM were cultured in medium only (mock), medium containing UV-inactivated ECTV (uvi-ECTV) or live-ECTV (ECTV) for 4, 12 and/or 24 h. In some experiments cells were left untreated or were additionally treated with LPS (1 μg/ml). (A) Representative images of mock–, uvi-ECTV–and ECTV–treated GM-BM at 4 and 24 hpi stained with Hoechst 33342 (blue fluorescence) and pAbs anti-ECTV (green fluorescence). The magnified images are of the boxed regions. Arrows indicate viral particles; arrowheads show viral factories. Scale bars = 10 μm. (B) Representative histograms showing the percentage of ECTV^+^ cells at 4, 12 and 24 hpi of GM-BM. Numbers represent the percentage of ECTV^+^ cells. (C) The mean percentage of ECTV^+^ cells during infection in GM-BM. Error bars represent ± SD from three independent experiments.(D) Scanning electron microscopy micrograph of GM-BM surface at 24 hpi. The magnified images are of the boxed regions. Arrows indicate viral particles. Scale bars = 2.5 μm. IsCon–isotype control.

GM-BM at early [4 hour post infection (hpi)] and late (24 hpi) stages of infection were labeled with anti-ECTV polyclonal antibodies (pAbs) and Hoechst 33342 to detect viral antigens or nuclear and viral dsDNA, respectively. At the early stage (4 hpi) of infection with live-ECTV, GM-BM displayed the presence of several viral replication centers, called “viral factories”. Such highly compartmentalized replication centers were visualized within the cytoplasm as regular small or ellipsoidal areas showing simultaneous labeling of dsDNA and viral proteins with Hoechst 33342 and anti-ECTV pAbs, respectively (Fig 1A). At the late stage (24 hpi) of infection with live-ECTV, majority of the infected cells had larger, but still regular viral factories and numerous progeny virions located near the viral factories and within cellular extensions, formed extensively by infected cells (Fig 1A). Presence of multiple cellular extensions gave the cells a unique, multifaceted morphology, previously observed in ECTV-infected L929 fibroblasts [27]. Moreover, at 24 hpi viral factories in infected GM-BM were strongly stained with anti-ECTV pAbs, suggesting that the quantity of antigens recognized by those antibodies increased together with the progress of virus replication. In contrast, at 4 and 24 hpi GM-BM treated with UV-inactivated (uvi)-ECTV exhibited only the presence of single virions on the cell surface and/or in the cytoplasm, without any visible signs of viral reproduction (Fig 1A), indicating complete inactivation of the virus.

Consistent with the above finding, intracellular staining with anti-ECTV pAbs and flow cytometry analysis revealed that the percentage of ECTV^+^ cells expressing high levels of viral antigens increased with the duration of infection (Fig 1B). At 4, 12 and 24 hpi the mean percentage of ECTV^+^ cells was 6%, 47% and 77%, respectively (Fig 1C). Moreover, in LPS-treated ECTV-infected GM-BM the percentage of ECTV^+^ cells was comparable with values obtained in LPS-untreated ECTV-infected cells and reached 8% at 4 hpi, rising to 41% and 78% at 12 and 24 hpi, respectively (Fig 1C). Collectively, the above data suggest that the tempo of viral reproduction in GM-BM is independent of the presence of TLR4-agonist. Moreover, a high percentage of ECTV^+^ cells at 24 hpi indicates that ECTV is able to replicate not only in macrophages, but also in cDCs found in GM-BM cultures. Meanwhile, in the population of GM-BM-treated with uvi-ECTV, similar to the uninfected cell population, no appearance of ECTV^+^ cells was observed during the course of infection.

The last stage of a productive viral replication cycle is the release of progeny virions from the infected cells. Accordingly, we performed a detailed analysis of the cell surface of ECTV-infected GM-BM using scanning electron microscopy (SEM). As determined by SEM, at 24 hpi numerous progeny virions remained attached singly or in clusters to the surface of the cellular membrane (Fig 1D). Probably, ECTV progeny virions were exposed on the GM-BM surface by exocytosis and were bound as cell-associated enveloped virus (CEV) [28].

To confirm that ECTV is able to replicate productively in cDCs we additionally infected the JAWS II cell line–a GM-CSF-dependent DC line [29]. At 24 hpi JAWS II cells exhibited typical signs of ECTV replication, i.e., presence of viral factories located in the perinuclear region, formation of long cellular extensions and presence of progeny virions near the plasma membrane and within cellular extensions (S2 Fig). Taken together, our data clearly indicate that ECTV, unlike VACV and CPXV, is able to productively replicate in cDCs.

### ECTV causes dramatic changes in morphology and induces apoptosis in GM-BM during late stages of infection

**Fig 2:**
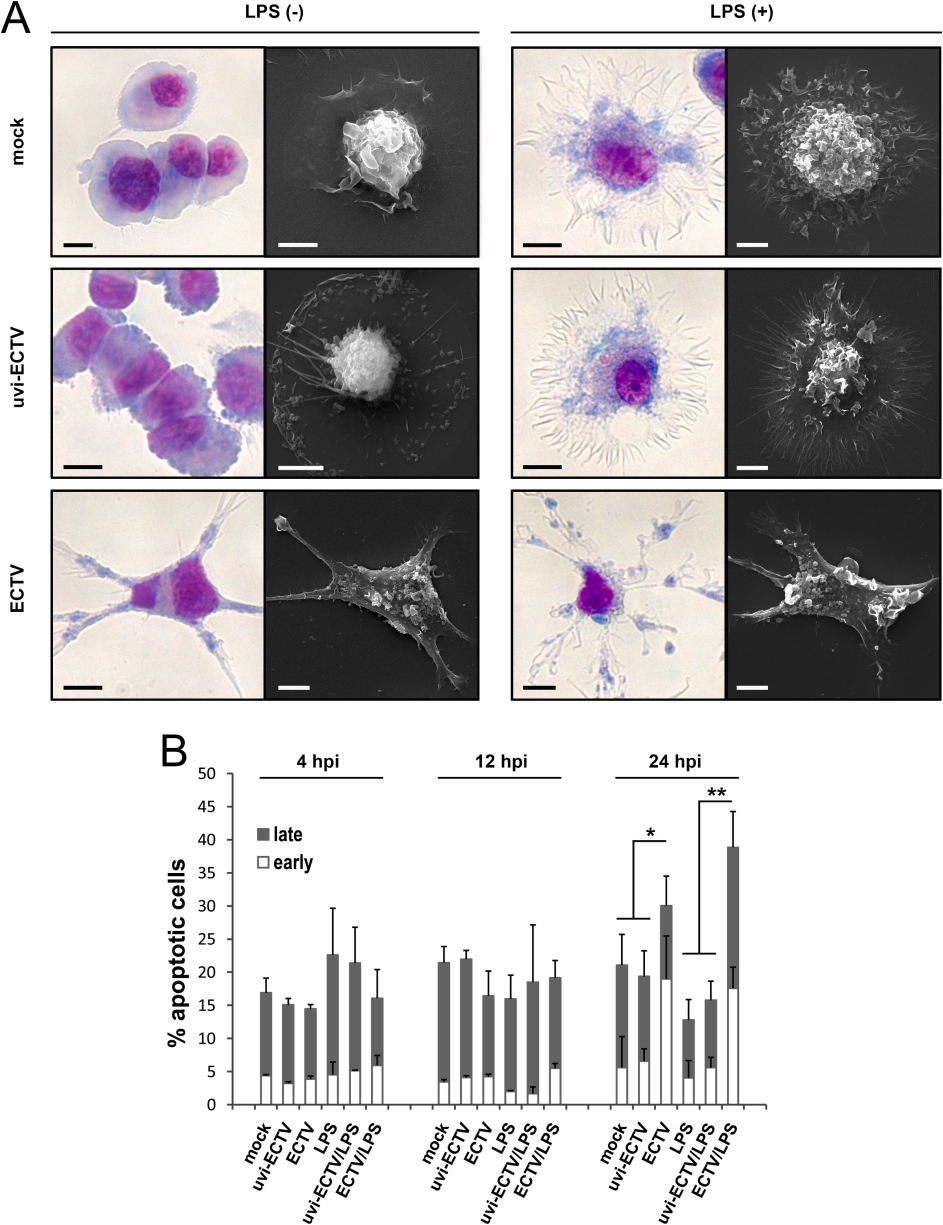
ECTV induces morphological changes and apoptosis in infected GM-BM. (A) Morphological changes of GM-BM infected with ECTV. GM-BM were cultured in medium only (mock), medium containing UV-inactivated ECTV (uvi-ECTV) or live-ECTV (ECTV) and were stimulated with or without LPS (1 μg/ml) for 24 h. Left panels show representative images of May-Grünwald-Giemsa stained GM-BM. Right panels demonstrate representative scanning electron microscopy micrographs of GM-BM. Scale bars = 5 μm. (B) The mean percentage of early and late apoptotic cells in GM-BM at 4, 12 and 24 hpi. Error bars represent ± SD from three independent experiments (Student’s *t*-test; ^*^P< 0.05, ^**^P< 0.01).

Because productive ECTV infection seems to affect cell shape of murine cDCs, we performed additional microscopic analysis of cellular morphology of mock-, uvi-ECTV- and ECTV-infected GM-BM, untreated or treated with LPS for 24h. Mock- and uvi-ECTV-exposed GM-BM exhibited morphology typical for immature DCs: high nucleus to cytoplasm ratio and the presence of short, blunt prolongations and/or few prominent dendritic projections (Fig 2A). Upon stimulation with LPS for 24 h (the time which is needed for full maturation of DCs), mock- and uvi-ECTV-treated GM-BM increased in size and became mature, what was associated with lower nucleus to cytoplasm ratio and appearance of abundant and well-developed dendrites (Fig 2A). In contrast, GM-BM infected with live-ECTV demonstrated totally different morphology. Infected cells lost the vast majority of their dendritic projections, but instead exhibited the presence of multiple long cellular extensions, which were numerous and irregular in infected LPS-treated GM-BM (Fig 2A). Moreover, infected cells showed cell surface flattening accompanied by gradual loss of veils. Taken together, our data demonstrate that GM-BM infected with ECTV exhibit a severely altered morphology and do not acquire morphological features of maturation after LPS stimulation.

Next, we asked whether the productive ECTV infection may affect cell viability and induce apoptosis in GM-BM. During the first 12 h of infection the total percentage of early and late apoptotic cells did not change between ECTV-infected- and mock-/uvi-ECTV-treated GM-BM incubated with or without LPS (Fig 2B). Meantime, at 24 hpi the percentage of early apoptotic cells significantly (*P* ≤ 0.05) increased in ECTV-infected GM-BM compared to mock-/uvi-ECTV-treated cells. Moreover, ECTV infection of GM-BM in the presence of LPS significantly (*P* ≤ 0.01) increased the percentage of both early and late apoptotic cells compared with cells treated with medium + LPS or uvi-ECTV + LPS (Fig 2B). These results indicate that ECTV induces apoptosis of GM-BM during the late stages of infection.

### GM-BM infected with ECTV exhibit reduced antigen uptake and processing ability

The productive ECTV infection that severely altered the morphology of GM-BM, cancertainly affect their innate and adaptive immune functions and/or maturation status. Therefore, we assessed immaturity *vs* maturity characteristics of ECTV-infected GM-BM in regard to antigen uptake and processing. The capture of antigen is the primary function of immature DCs.

**Fig 3:**
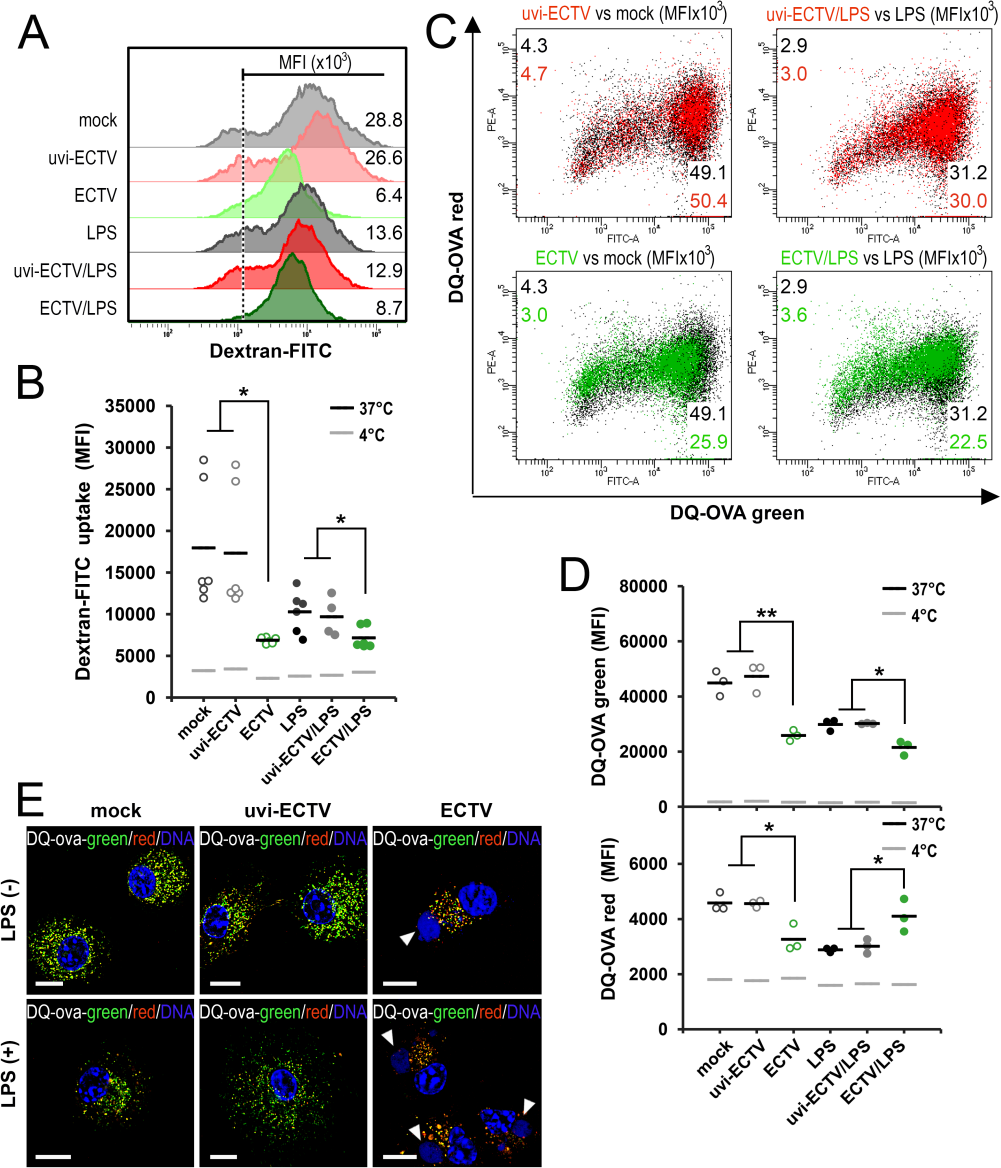
GM-BM infected with ECTV have reduced capacity to capture and process exogenous antigen. Mock-, uvi-ECTV- or ECTV-infected GM-BM untreated or treated with LPS for 24 h were pulsed with 70 kD dextran-FITC (0.5 mg/ml) or DQ-OVA (30 μg/ml) for 60 min at 4°C and 37°C, and analyzed by flow cytometry. (A) Representative histograms of dextran-FITC uptake by GM-BM, which is expressed as mean fluorescence intensity (MFI). (B) Graph represents individual data of MFI for dextran-FITC uptake at 37°C from at least three independent experiments (paired Student’s *t*-test; ^*^P< 0.05). Black and grey lines indicate the mean values of dextran-FITC uptake at 37°C and 4°C, respectively. (C) Representative overlay dot plots showing DQ-OVA processing by uvi-ECTV- or ECTV-infected GM-BM *vs* mock-infected cells, and GM-BM treated with uvi-ECTV + LPS or ECTV + LPS *vs* LPS alone. Numbers represent the MFI for DQ-OVA green fluorescence (X axis) and DQ-OVA red fluorescence (Y axis). (D) Graph represents individual data of MFI for DQ-OVA red and DQ-OVA green at 37°C from three independent experiments. Black and grey lines indicate the mean values of DQ-OVA red and DQ-OVA green at 37°C and 4°C, respectively (paired Student’s *t*-test; ^*^P< 0.05, ^**^P< 0.01). (E) Fluorescence microscopy images depict DQ-OVA processing (green fluorescence) and accumulation of digested DQ-OVA fragments at a high concentration in organelles (red fluorescence) in GM-BM. Nuclear and viral DNA is stained with Hoechst 33342. Arrowheads indicate viral factories. Scale bar = 10 μm.

GM-BM were assessed for uptake of the FITC-labeled dextran, which is predominantly taken up by nonspecific pinocytosis (macropinocytosis), but can also be internalized through receptors [30], and DQ-OVA, which uses the mannose receptor for endocytosis into cells. Unstimulated mock- and uvi-ECTV-treated GM-BM at 24 hpi showed the strongest capability to capture FITC-dextran with low nonspecific binding (Fig 3A and 3B). Stimulation of GM-BM with LPS for 24 h resulted in inhibition of the ability to take up FITC-dextran, what was consistent with the hallmark of maturation. A significant (*P* ≤ 0.05) decrease of FITC-dextran mean fluorescence intensity (MFI) was detected in ECTV-infected cells at 24 hpi, compared with mock- and uvi-ECTV-treated cells. Similarly, cells treated with ECTV + LPS exhibited a significant reduction of FITC-dextran uptake, compared with cells incubated with medium + LPS and uvi-ECTV + LPS (Fig 3B). These results indicate, that antigen uptake by nonspecific endocytosis is severely reduced in ECTV-infected GM-BM.

To further characterize the influence of ECTV-infection on the receptor-mediated endocytosis and antigen processing in GM-BM we used ovalbumin (OVA) labeled with the pH-insensitive fluorescent dye (FL) boron-dipyrromethene (BODIPY)–DQ-OVA, as a soluble antigen. DQ-OVA is endocytosed *via* the mannose receptor. After endocytosis, DQ-OVA conjugate is digested by the endo-lysosomal proteases and released fragments of DQ-OVA exhibit bright-green fluorescence. The extent of DQ-OVA processing is proportional to the level of fluorescence, and this corresponds to the extent of receptor-mediated internalization within the cell [31]. In addition, when the digested fragments of DQ-OVA accumulate in organelles at high concentration, the BODIPY FL fluorophore may form excimers that exhibit red fluorescence [32].

Our data revealed that in mock- or uvi-ECTV-treated GM-BM the largest amount of DQ-OVA was endocytosed and degraded at 24 hpi, as indicated by the high MFI value for green fluorescence (Fig 3C and 3D). Moreover, in these cells OVA accumulated at high densities, as determined by the high MFI value for red fluorescence. Treatment of mock- or uvi-ECTV-infected GM-BM with LPS for 24 h resulted in their maturation, since DQ-OVA degradation and its accumulation at high concentration in cells were inhibited. A similar trend was observed in ECTV-infected GM-BM, where the mean values of MFI for both green and red fluorescence significantly (*P* ≤ 0.01) decreased compared with MFI values for mock- or uvi-ECTV-treated cells. Meanwhile, GM-BM treated with ECTV + LPS showed a decreased rate of soluble antigen degradation (green fluorescence) and increased OVA accumulation (red fluorescence) compared to GM-BM treated with medium + LPS or uvi-ECTV + LPS (Fig 3C and 3D). The decreased ability of DQ-OVA processing by infected cells was additionally confirmed by fluorescence microscopy, which indicated that the green fluorescence disappeared almost completely in ECTV-treated cells (Fig 3E). Taken together, these findings establish that ECTV reduces capacity of GM-BM to endocytose and process a soluble antigen and treatment with LPS modifies antigen processing in infected GM-BM.

### ECTV infection inhibits full maturation of GM-BM

**Fig 4:**
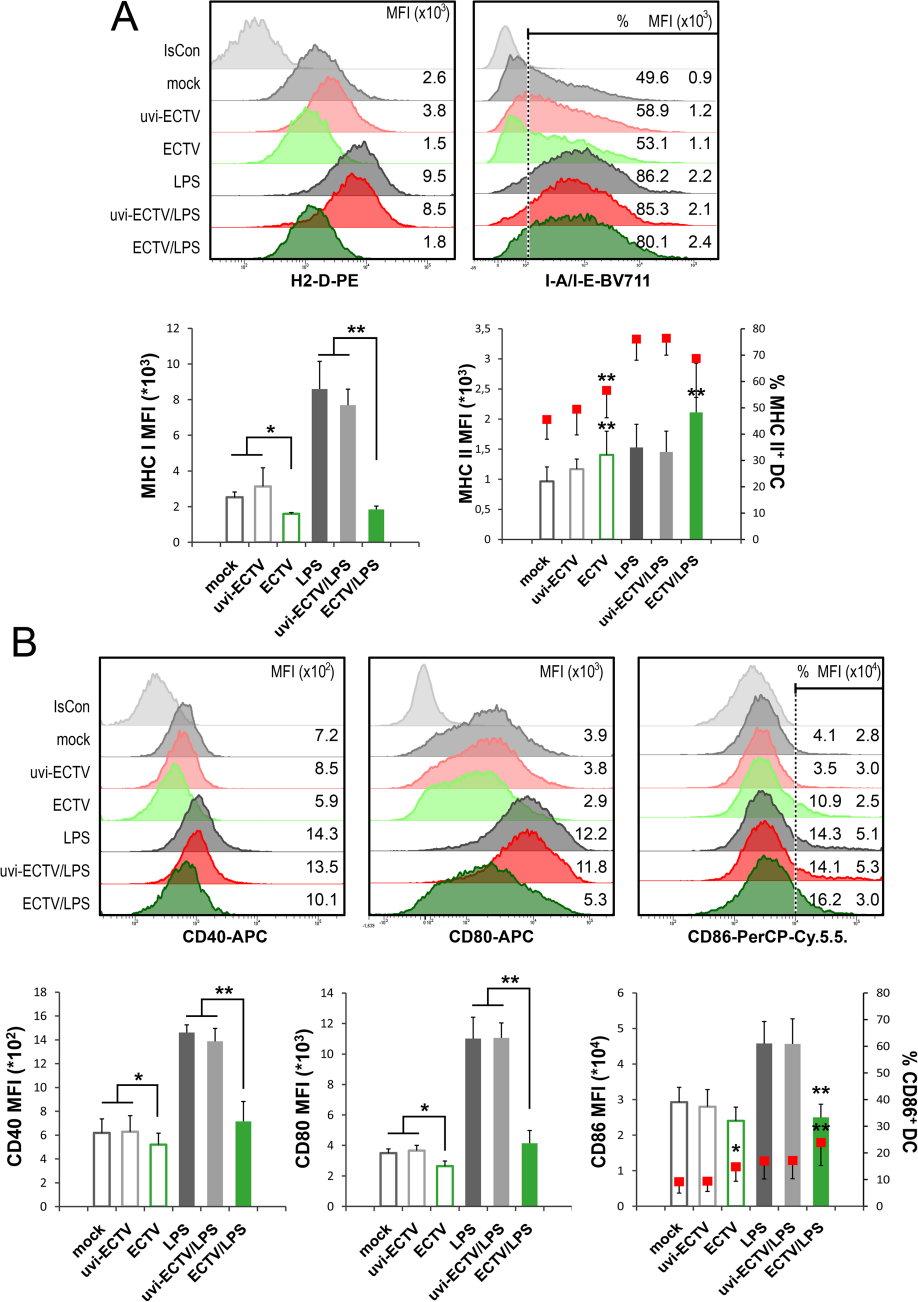
ECTV infection does not trigger phenotypic maturation and inhibits LPS-induced maturation of GM-BM. Mock-, uvi-ECTV- or ECTV-infected GM-BM were left untreated or were treated with LPS for 24 h. Representative histograms showing the major histocompatibility complex (MHC) (A) or co-stimulatory (B) molecules expression on GM-BM. Numbers represent the MFI value and/or the percentage of positive cells for a given marker. Graphs show mean ± SD of MFI and/or percentage for indicated marker from at least three independent experiments (paired Student’s *t*-test; ^*^P< 0.05, ^**^P< 0.01). Statistical comparisons were between mock- or uvi-ECTV-treated DCs and ECTV-exposed DCs and between LPS- or uvi-ECTV+LPS-treated DCs and ECTV + LPS-exposed DCs. IsCon–isotype control.

Since ECTV-infected GM-BM lost their antigen*-*capturing and -processing ability, as generally occurs when DCs differentiate into mature cells, our next question concerned the influence of ECTV infection on GM-BM phenotypic maturation. We analyzed the expression of MHC class I and MHC class II molecules, as well as co-stimulatory molecules: CD40, CD80 and CD86 on GM-BM after 24 h exposure of cells to medium, uvi-ECTV or live-ECTV, in the presence or absence of LPS. The MFI of H-2D and I-A/I-E increased on mock- and uvi-ECTV-infected GM-BM treated with LPS for 24 h, as compared to untreated cells (Fig 4A). ECTV-infected and ECTV + LPS-treated cells showed significantly (*P* ≤ 0.05) reduced MFI of H-2D and significantly (*P* ≤ 0.01) elevated MFI of I-A/I-E compared with cells exposed to mock and mock + LPS, respectively. Moreover, the percentage of I-A/I-E-positive cells was significantly (*P* ≤ 0.01) increased in ECTV-exposed compared to mock-exposed cells (Fig 4A). Thus, the increase in the percentage of MHC II-positive cells and the elevated level of MHC II expression is observed in ECTV-infected GM-BM.

The expression of all analyzed co-stimulatory molecules was significantly (*P* ≤ 0.05) reduced on GM-BM infected with ECTVcompared to mock- or uvi-ECTV-treated cells (Fig 4B). Moreover, ECTV-infected GM-BM treated with LPS were unable to increase their expression of CD40, CD80 and CD86 molecules, and the inhibitory effect of the virus was more spectacular when compared between cells treated with ECTV + LPS and cells exposed to mock + LPS or uvi-ECTV + LPS. Despite the reduction of CD86 level on ECTV-infected cells, the percentage of CD86-positive cells was significantly (*P* ≤ 0.01) higher in GM-BM infected with ECTV, compared to mock- or uvi-ECTV-treated cells, cultured with or without the TLR4-agonist. Taken together, our data suggest that upon infection with ECTV GM-BM are unable to fully mature but retain immature phenotype and therefore may fail to stimulate a protective antiviral immune response.

### GM-BM infected with ECTV fail to produce cytokines and chemokines in response to TLR4-agonist treatment

In addition to the shift in endocytic activity and phenotype, maturation of DCs is also associated with changes in cytokine and chemokine production, which are pivotal in activation and polarization of Th immune response and further determine the outcome of infection and disease severity. Therefore, we evaluated the production of pro-inflammatory cytokines and chemokines involved in T cell priming/activation (TNF-α and IL-6) and engaged in mounting Th1 [IL-12p40, IL-12p70, CCL3/macrophage inflammatory protein 1 alpha (MIP-1α) and CCL5/regulated upon activation normal T cell expressed and secreted (RANTES)] or Th2 [IL-10 and CCL2/monocyte chemoattractantprotein 1(MCP-1)] polarization.

**Fig 5:**
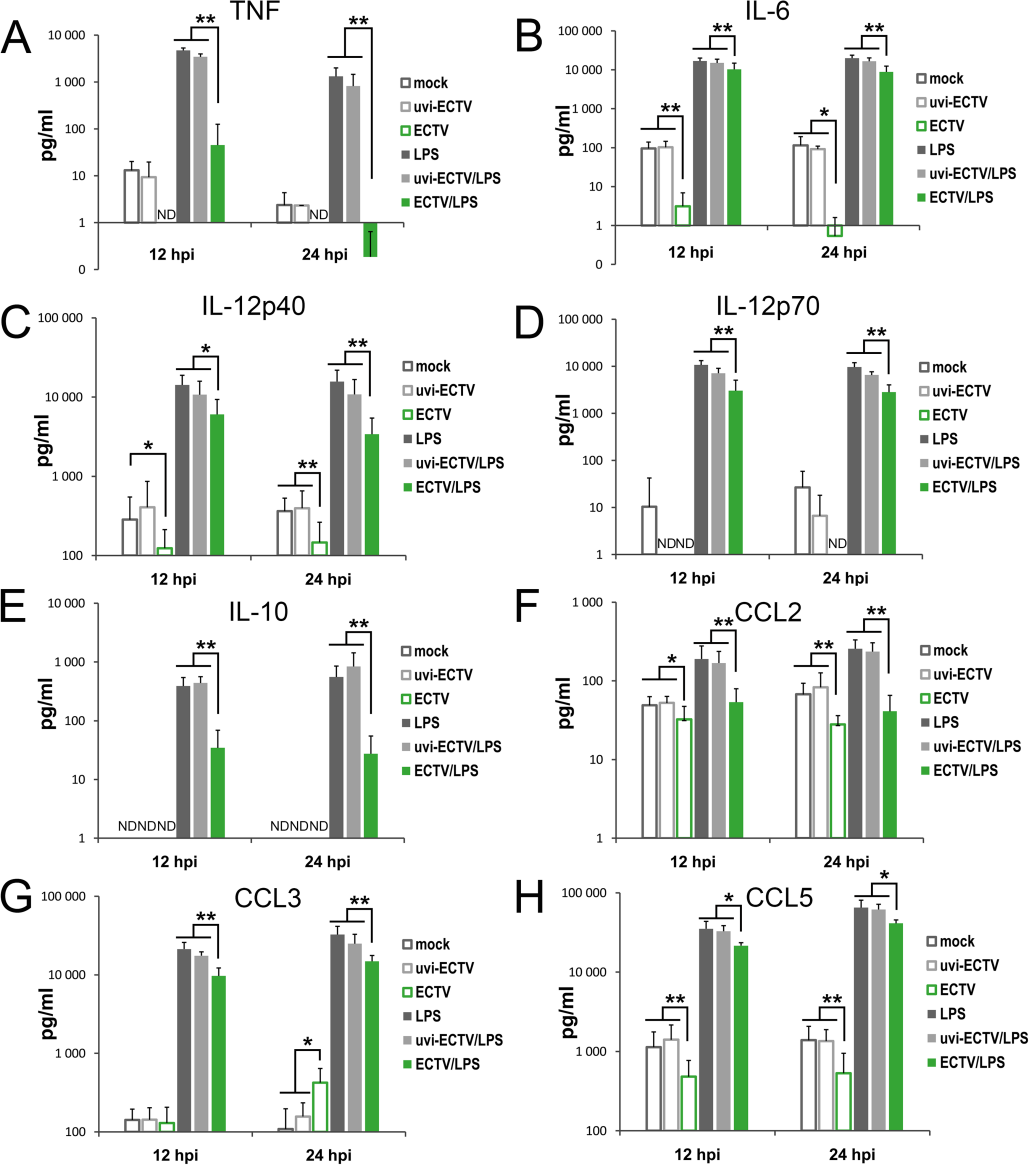
ECTV infection alters cytokine and chemokine production by GM-BM. Culture supernatants from mock-, uvi-ECTV- or ECTV-infected GM-BM untreated or treated with LPS for 12 and 24 h were analyzed for the presence of TNF-α (A), IL-6 (B), IL-12p40 (C), IL-12p70 (D), IL-10 (E), CCL2/MCP-1 (F), CCL3/MIP-1α (G) and CCL5/RANTES (H). Data are shown as mean ± SD from at least three independent experiments (paired Student’s *t*-test; ^*^P< 0.05, ^**^P< 0.01). ND–not detected.

Our results showed that after 12 and 24 hpi ECTV-infected GM-BM secreted less pro-inflammatory cytokines, such as: TNF-α (Fig 5A), IL-6 (Fig 5B), IL-12p40 (Fig 5C) and IL-12p70 (Fig 5D), and chemokines CCL3/MCP-1 (Fig 5G) and CCL5/RANTES (Fig 5H) compared with mock- or uvi-ECTV-treated cells. ECTV infection did not stimulate IL-10 production (Fig 5E), but induced CCL3/MIP-1α secretion by GM-BM at 24 hpi (Fig 5G). In turn, LPS treatment induced robust production of all analyzed cytokines and chemokines. Meanwhile, ECTV-infected GM-BM treated with LPS were able to secrete those cytokines, but the amounts were significantly (*P* ≤ 0.05) lower compared with those secreted by mock- or uvi-ECTV-infected LPS-treated GM-BM (Fig 5). Taken together, our results indicate that ECTV significantly influences the production of cytokines and chemokines involved in regulation of T cell response by GM-BM through inhibition of TLR signaling pathway.

### ECTV infection inhibits nuclear translocation of NF-κB, IRF3 and IRF7 in GM-BM

Because ECTV infection altered TLR4-induced secretion of cytokines and chemokines, next we asked whether such inhibitory effect was caused by prevention of nuclear translocation of key TLR-dependent transcription factors, including NF-κB, IRF3 and IRF7. Those transcription factors are coordinately activated through TLR4 ligands and thereby activate gene expression and trigger antiviral immune response.

**Fig 6:**
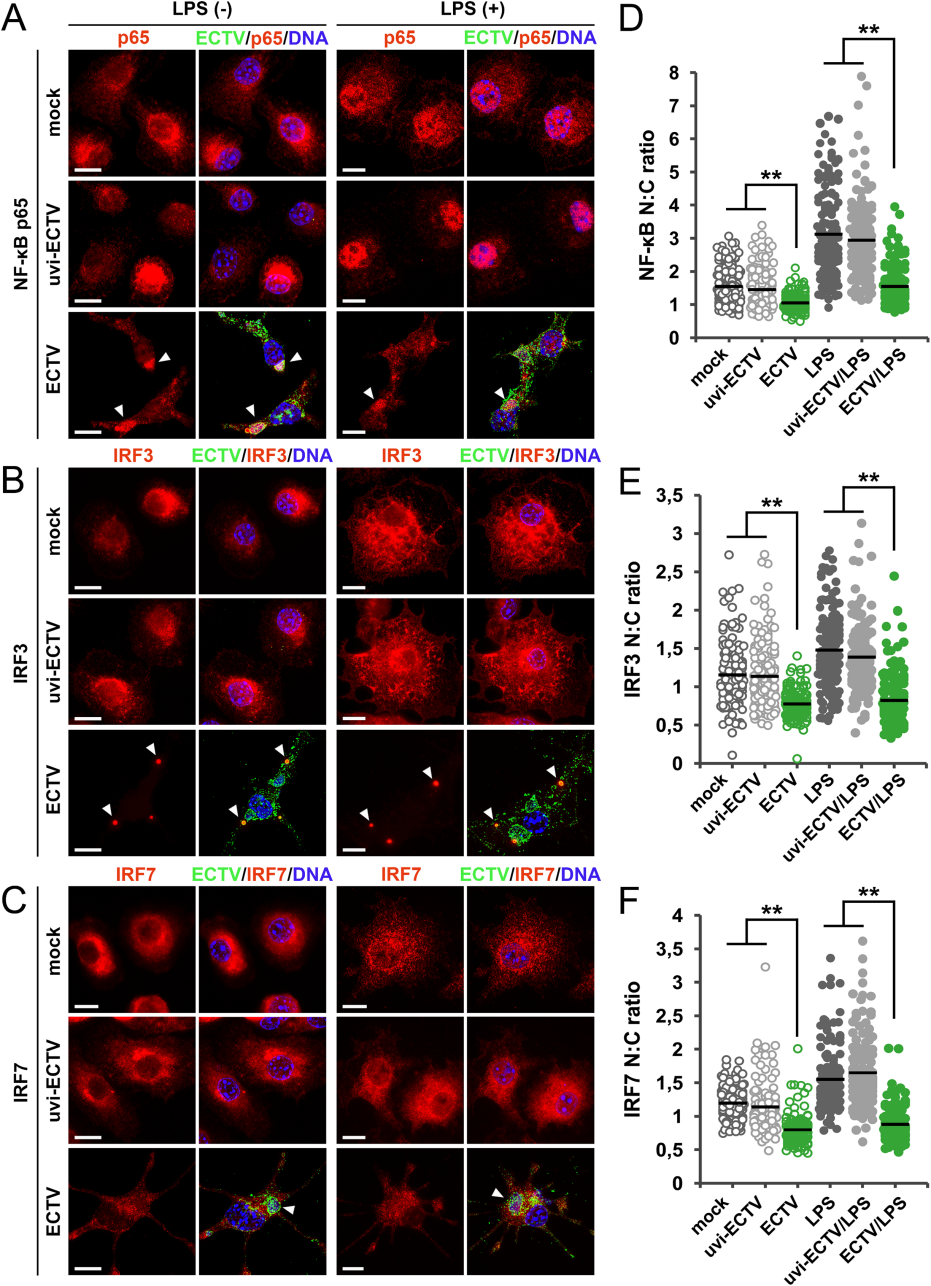
ECTV infection inhibits nuclear translocation of NF-κB, IRF3 and IRF7 in GM-BM. Indirect immunofluorescence analysis of nuclear translocation of NF-κB (A), IRF3 (B) and IRF7 (C) in mock-, uvi-ECTV- or ECTV-infected GM-BM untreated or treated with LPS for 24 h. Left panels show images of red fluorescence channel for the indicated transcription factor, right panel show images of merge fluorescence channels for the indicated transcription factor (red), viral antigen (green), and nuclear and viral DNA (blue). Arrowheads show viral factories (A, C) or vacuoles (B) in infected cells. Scale bars = 10 μm. Nuclear [N]: cytoplasmic [C] ratios of NF-κB (D), IRF3 (E) and IRF7 (F) were determined at single cell level by measuring of fluorescence signal intensities within the nucleus (stained with Hoechst 33342) and the cytosol. Analysis was performed on 50 cells/condition and experiment (from three independent experiments). Black lines indicate the mean values of each data set (Student’s *t*-test;^**^P< 0.01).

Nuclear translocation and retention of NF-κB/p65, IRF3 and IRF7 in mock-, uvi-ECTV- and ECTV-infected GM-BM was analyzed in response to stimulation with the TLR4 agonist–LPS. Mock-, uvi-ECTV- and ECTV-infected cells were cultured in the presence or absence of LPS and after 24 h intracellular localization of p65, IRF3 and IRF7 was evaluated by fluorescence microscopy using primary antibodies specific for the indicated proteins and Rhodamine RedX-conjugated secondary antibodies. In ECTV-infected GM-BM the nuclear presence of p65, IRF3 and IRF7 was significantly (*P* ≤ 0.01) decreased compared with mock- or uvi-ECTV-treated cells (Fig 6). Treatment of mock- and uvi-ECTV-infected cells with LPS enhanced p65 and IRF7 translocation to the nucleus, whereas nuclear to cytoplasmic ratio of IRF3 was only slightly increased. On the other hand, ECTV-infected GM-BM treated with LPS showed significantly (*P* ≤ 0.01) decreased nuclear translocation of p65, IFR3 and IRF7 compared with cells incubated with medium + LPS and uvi-ECTV + LPS (Fig 6). Moreover, fluorescence microscopy analysis revealed that p65 co-localized with viral factories in infected cells, independently of LPS influence (Fig 6A). Cytosolic localization of IRF3 was diminished and this transcription factor was found predominantly and almost exclusively in the cytoplasmic vacuoles formed in ECTV-infected GM-BM (Fig 6B). Taken together, our data indicate that infection of GM-BM with ECTV inhibits nuclear translocation and sustained retention of NF-κB/p65, IRF3 and IRF7 in the nucleus induced by LPS treatment.

### ECTV infection impairs the expression of genes involved in TLR, RLR, NLR and IFN type I signaling pathways in GM-BM

**Fig 7:**
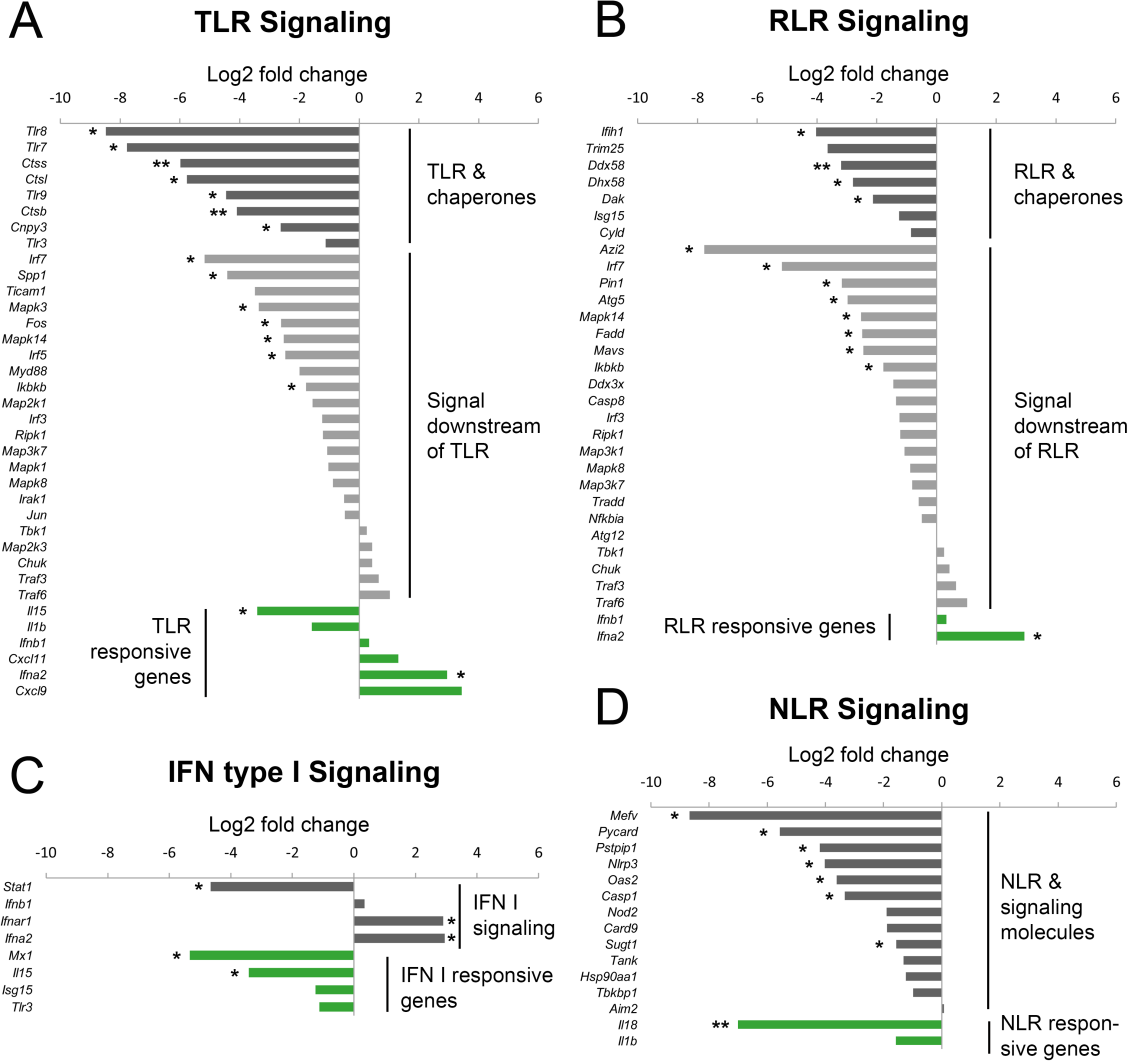
ECTV infection modulates the expression of genes engaged in the innate antiviral immune response. Log2 fold change of mRNA expression for genes associated with Toll-like receptor (TLR) (A), RIG I-like receptor (RLR) (B), IFN type I (C) and NOD-like receptor (NLR) (D) signaling. Average threshold cycle (C_T_) values from PCR reactions were normalized against the average C_T_ values for the endogenous control *Atg12* from the same cDNA sample and shown as 2^(-ΔΔCT) were ΔC_T_ = C_T gene_− C_T atg12_. Graph columns represent the mean values of log2 fold change in mRNA expression from two independent experiments (Student’s *t*-test; ^*^P< 0.05, ^**^P< 0.01).

Suppression of nuclear translocation of NF-κB/p65, IRF3 and IRF7 may be an explanation of reduced pro-inflammatory cytokine secretion by GM-BM infected with ECTV. However, transcription of genes that encode pro-inflammatory cytokines and type I IFNs is mediated by signaling pathways that are activated after recognition of viral components by different pattern recognition receptors (PRRs), such as TLR, RLR and NLR. For that reason, we assessed whether ECTV infection of GM-BM alters transcription of selected genes involved in TLR, RLR, NLR and IFN signaling pathways. Analyzed genes were divided into three functional groups. The first group represented receptor and chaperone genes, the second involved genes in the downstream signaling and the third–responsive genes (Fig 7). Genes in each of the data sets were deemed reflective of changes in mRNA transcription if they showed at least a two-fold change in expression.

In the chaperone and receptor group of the TLR signaling category, ECTV significantly (*P* ≤ 0.05) down-regulated of *Tlr7*, *Tlr8*, *Tlr9* mRNA and genes for cathepsin B, L and S (Fig 7A). Moreover, *Cnpy3*, encoding TLR-specific co-chaperone for HSP90B1, which mediates the proper folding and maturation of TLR and hence controls TLR exit from the endoplasmic reticulum (ER), was also down-regulated in infected GM-BM. In the TLR downstream signal group, *Irf5*, *Irf7*, mitogen-activated protein kinase (MAPK) genes *Mapk3* and *Mapk14*, and other genes involved in MyD88-dependent TLR signaling, such as *Spp1* and *Fos* were significantly down-regulated in ECTV-infected GM-BM (Fig 7A). Additionally, the expression of *Ikbkb* encoding inhibitor of NF-κB (IκB) kinase β (IKKβ) that phosphorylates IκB causing its degradation and subsequent activation of NF-κB, was also significantly (*P* ≤ 0.05) decreased. Furthermore, ECTV induced down-regulation of *Il15* and up-regulation of type I IFN gene *Ifna2* in the group of the TLR-responsive genes. Type I IFNs play a crucial role in nonspecific defense against viruses, however, orthopoxviruses such as VACV, VARV, MPXV and ECTV, encode a Type I IFN-binding protein that acts as a decoy receptor for type I IFN and blocks IFN-α transmembrane signaling [33].

In the RIG-I-like receptor signaling category 4 genes in the receptor and chaperone group were down-regulated in ECTV-infected GM-BM: *Ifih1*, *Ddx58*, *Dhx58* and *Dak* (Fig 7B), encoding MDA5, RIG-I, LGP2 and DAK, respectively. Meanwhile, in the group of 22 genes involved in the RLR downstream signaling, ECTV infection of GM-BM resulted in down-regulation of 8 genes: *Azi2*, *Irf7*, *Pin1*, *Atg5*, *Mapk14*, *Fadd*, *Mav*s and *Ikbkb*. In the responsive gene group, only *Ifna2* was significantly (*P* ≤ 0.05) up-regulated whereas the expression of *Ifnb1* remained unchanged in infected cells.

Two groups of genes were analyzed in the IFN type I signaling category (Fig 7C). In the group of IFN I signaling, *Stat1* was significantly (*P* ≤ 0.05) down-regulated whereas *Ifnar1* and *Ifna2* were significantly (*P* ≤ 0.05) up-regulated in ECTV-infected GM-BM. Meanwhile, in the group of IFN I responsive genes, *Mx1* and *Il15* were down-regulated whereas the expression of *Isg15* and *Tlr3* was unchanged. A profound inhibitory influence of the virus was observed in the NLR signaling category, since ECTV significantly (*P* ≤ 0.05) down-regulated 10 of 15 analyzed genes (Fig 7D). In the group of NLR and signaling molecules, ECTV decreased the expression of *Mefv*, *Pycard*, *Pstpip1*, *Nlrp3*, *Oas2*, *Casp1*, *Nod2*, *Card9* and *Sugt1*, whereas in the group of NLR responsive genes ECTV significantly (*P* ≤ 0.05) down-regulated the expression of *Il18*.

Taken together, our results clearly indicate that ECTV impairs numerous genes involved in the TLR, RLR, IFN type I and NLR signaling pathways, required for induction of an antiviral immune response in infected GM-BM. ECTV-mediated down-regulation of genes engaged in multiple PRR signaling pathways occurs at different stages, including initial genes of receptors and chaperones, intermediate genes of downstream signaling events and late responsive genes. These data provide molecular evidence, that a single virus is able to affect numerous PRR signaling genes to down-regulate innate and adaptive immune responses in murine dendritic cells.

### GM-BM infected with ECTV failed to stimulate proliferation of allogeneic CD4^+^ T cells

**Fig 8:**
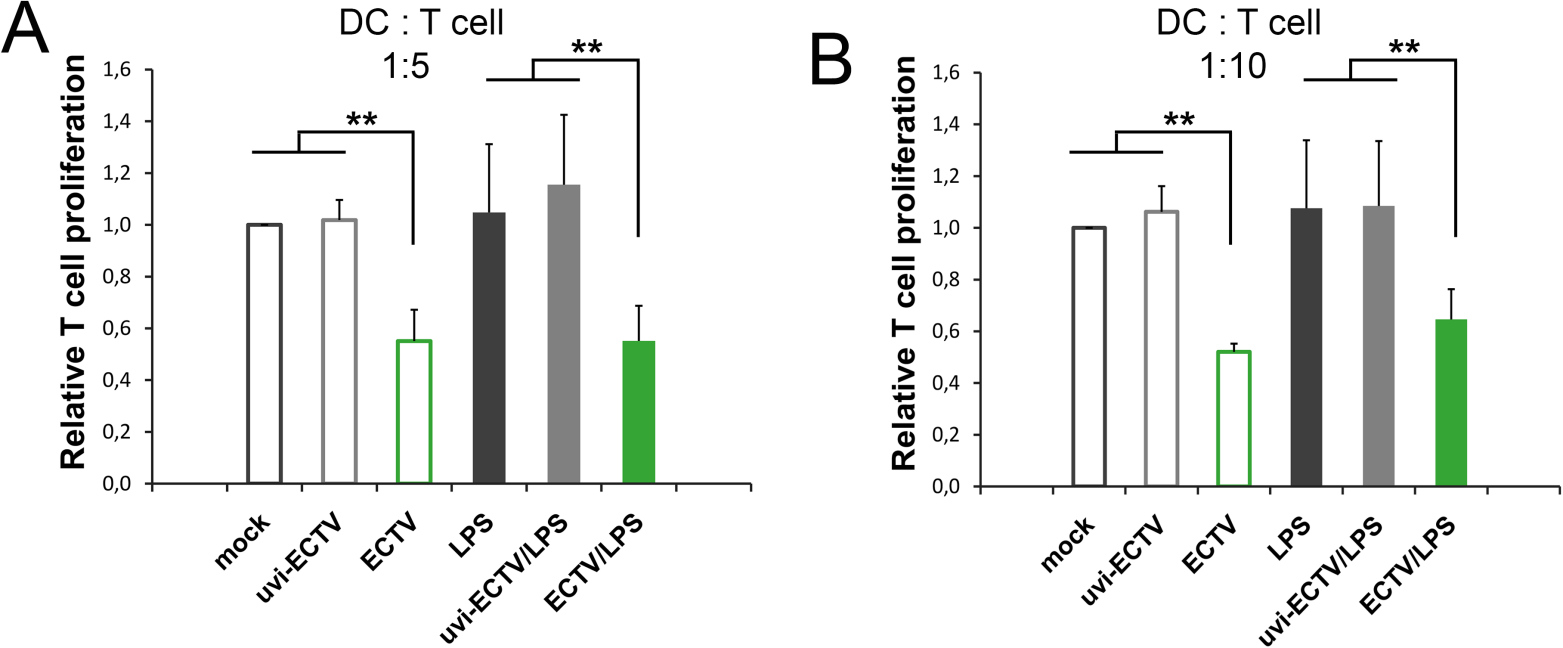
GM-BM infected with ECTV exhibit impaired capacity to stimulate allogeneic CD4^+^ T cell proliferation. Mock-, uvi-ECTV- or ECTV-infected GM-BM, untreated or treated with LPS for 24 h, were co-cultured for five days with allogeneic CD4^+^ T cells at 1:5 and 1:10 DC/T cell ratio. Cells were incubated for additional 2 h with WST-8. Data were normalized to the values obtained in mock-infected GM-BM. Graphs represent mean ± SD from three independent experiments (paired Student’s *t*-test; ^*^P< 0.05, ^**^P< 0.01).

Our last question concerned the capacity of ECTV-infected GM-BM to stimulate T cell immune response. To characterize adaptive functional properties of GM-BM infected with the virus, we assessed weather those cells are able to stimulate proliferation of purified allogeneic CD4^+^ T cells from C57BL/6 mice in a primary mixed leukocyte reaction (MLR). GM-BM incubated in medium or treated with uvi-ECTV stimulated allogeneic CD4^+^ T cell proliferation similar to cells additionally treated with LPS (Fig 8). Meanwhile, ECTV-infected GM-BM, untreated or treated with LPS, were ineffective in stimulating T cell proliferation when co-cultured with T cells in a ratio of 1:5 or 1:10. Our data indicate that ECTV, like other orthopoxviruses, such as cowpox virus (CPXV), impairs DCs function to stimulate proliferation of T cells [22].

## Discussion

Since ECTV is a highly host specific virus, with the host range restricted to mouse, in the present study we evaluated the effect of ECTV infection on innate and adaptive immunity properties of murine GM-BM, including cDCs. We demonstrated for the first time that ECTV is able to productively infect GM-BM *in vitro*, leading to their apoptosis. At later stages of infection numerous progeny virions appeared within the cytoplasm and on the cell surface, indicating their release from the infected cells. Moreover, the percentage of ECTV^+^ GM-BM expressing high levels of viral antigens increased with the duration of infection. Previously, we found that *in vivo* ECTV antigens are expressed by different APC subsets, including CD11c^+^, CD11b^+^ and CD19^+^ cells in secondary lymphoid organs (SLOs) of BALB/c and C57BL/6 mice after footpad infection [34]. Recently, ECTV was shown to infect multiple populations of DCs in draining lymph nodes of C57BL/6 mice, including conventional CD8α^+^ DCs and CD11b^+^ DCs and plasmacytoid DCs (pDCs) [35]. Additionally, infection of splenic DCs has been demonstrated to be persistent after acute mousepox in BALB/c mice, since DCs actively produced ECTV particles up to 60 days post-inoculation [36].

Contrary to ECTV, other orthopoxviruses, such as VACV and CPXV, cause nonproductive infection in DCs of human origin. VACV replicates abortively in both, immature and mature monocyte-derived DCs (MDDCs), but induces delayed apoptotic cell death only in the immature cell population [15]. VACV infection of MDDCs is characterized by the expression of early, but not late, viral-encoded proteins [24] and aborts before formation of replication centers, thus synthesis and accumulation of viral DNA do not occur [37]. In the case of CPXV, infection of cDCs, pDCs and MDDCs is nonproductive, but does not trigger cell death, and results in up-regulation of early viral immunomodulatory genes [22]. The difference between ECTV and other orthopoxviruses regard to the replication cycle in DCs may result from the highly specific restriction and adaptation of ECTV to its natural host. It is not excluded that ECTV has evolved unique strategies to overcome host immune defense, thus has more extensive control over DCs properties. Drillien et al. have proposed that abortive infection of VACV in DCs may be related to deficiency of DCs in the cellular protein factors or nucleotides necessary for orthopoxvirus DNA synthesis, due to terminal differentiation of these cells [24]. However, since ECTV productively infects DCs, it is possible that other factors than the terminal differentiation of DCs may be responsible for VACV or CPXV abortive replication in these cells [37].

ECTV infection of GM-BM had a significant effect on their morphology. Infected cells exhibited multiple irregular long cellular extensions, similar to those previously observed in ECTV-infected L929 fibroblasts [27]. Although VACV and CPXV have been shown to induce morphological changes of DCs, these viruses do not stimulate formation of long cytoskeletal protrusions in infected cells [22,38]. Abortive infection and lack of late viral gene expression may explain inability of VACV- and CPXV-infected DCs to generate such extensions, since formation of long-branched cellular projections depends on late viral protein synthesis [39].

GM-BM infected with ECTV displayed not only dramatic changes in morphology, but also exhibited multifunctional impairment in their innate and adaptive immune properties. The ability to engulf exogenous antigen was drastically reduced in ECTV-infected GM-BM. Moreover, their capacity to degrade soluble antigen was also severely impaired. Additionally, the surface expression of MHC class I molecules was decreased, however the expression of MHC II molecules was increased on infected GM-BM. Recently, it has been shown that endogenous MHC class II presentation pathway is active in DCs infected with MVA [40]. However, numerous studies have been shown that CPXV and VACV affect MHC class II [16,18,22] and MHC class I [41] antigen presentation pathways in DCs. CPXV inhibits MHC class I expression by two distinct mechanisms that act synergistically. Firstly, endogenous viral protein CPXV203 –retains peptide-loaded MHC class I molecules in the ER by exploiting the KDEL-mediated ER retention pathway. Secondly, another viral protein–CPXV12 –abrogates ER peptide loading and dissociation of MHC class I molecules from transporter associated with antigen processing (TAP) [42]. CPXV12 prevents peptide transport by inhibiting ATP binding to the nucleotide binding domains (NDBs) of TAP [43]. VACV inhibits MHC II expression on DCs and blocks presentation of antigenic peptides in MHC class II molecules by viral A35 protein, which localizes to endosomes [18,19]. Moreover, VACV reduces the level of lysosomal proteases and Ii degradation in infected APCs [44].

ECTV infection of GM-BM induced down-regulation of activation/maturation markers, such as CD40, CD80 and CD86, rendering those cells unable to fully mature upon stimulation with LPS. The reduced expression of CD86 on GM-BM infected with ECTV was correlated with an increased percentage of CD86^+^ population, independent of LPS stimulation. Other orthopoxviruses, such as VACV and CPXV have been shown to suppress maturation of DCs. VACV inhibits the expression of many activation markers, including CD83, CD86, HLA-DR and CD25 on human immature MDDCs cultured in the presence of maturation-inducing stimuli [15,45]. During LPS treatment, CPXV was shown to decrease the percentage of human MDDCs, cDCs and pDCs expressing CD80 and CD86, and additionally the percentage of cDCs and pDCs expressing CD40 and blood dendritic cell antigen 2 (BDCA2), respectively [22]. Taken together, our data are consistent with previous observations that orthopoxviruses severely affect maturation of cDCs in *in vitro* conditions.

Infection of GM-BM with ECTV did not stimulate cytokine and chemokine production, with the exception of CCL3/MIP-1α at later stages of infection. Possibly, the uninfected bystander DCs were the main source of CCL3/MIP-1α. It has been demonstrated that during MVA infection bystander DCs undergo maturation and are able to produce large amounts of a T cell-activating cytokine–IP-10/CXCL10 [46]. The production of cytokines by ECTV-infected GM-BM was also evaluated in response to LPS. LPS-TLR4 signaling involves Toll–IL-1–resistance (TIR) domain–containing adaptor–inducing IFN-β (TRIF) and TRIF-related adaptor molecule (TRAM) [47]. We showed that in response to LPS ECTV-infected GM-BM were able to secrete all tested cytokines, however, their production was severely decreased compared with mock-, or uvi-ECTV-treated cells cultured in the presence of the TLR4 agonist. Similar to ECTV, VACV infection of murine GM-BM fails to induce secretion of inflammatory cytokines [48]. Moreover, VACV has been shown to globally reduce production of cytokines and chemokines by APCs and responding T cells [18]. Similarly, CPXV infection of human MDDCs, cDCs and pDCs inhibits secretion of more than 20 cytokines and chemokines in response to TLR agonist treatment [22]. Taken together, our data are consistent with observations that orthopoxviruses suppress production of numerous cytokines and chemokines involved in regulation of innate and adaptive immune responses by cDCs.

Several possible mechanisms may explain the reduced synthesis of proinflammatory cytokines and chemokines by ECTV-infected GM-BM. First, we found that LPS-induced nuclear translocation of key transcription factors,such as NF-κB, IRF3 and IRF7 that control the transcription of cytokine and chemokine genes was impaired in ECTV-infected GM-BM. ECTV has been demonstrated to modulate NF-κB signaling pathway in other APCs [49], as well as in non-immune cells [50–52]. Inhibition of NF-κB activation can be mediated by ECTV-encoded ankyrin/F-box proteins, such as EVM002, EVM005, EVM159 and EVM170 that impair IĸBα degradation [53]. Moreover, ECTV encodes counterparts of the following VACV inhibitory proteins altering NF-κB signaling pathway: A46 that disrupts TLR and IL-1β signaling, B14 that targets the IκB kinase (IKK) complex, and N1 that probably acts upstream of IKK complex activation [49,51]. Interestingly, our studies revealed that p65 NF-κB is recruited to ECTV replication sites in GM-BM. On the contrary, p65 NF-κB has been shown to not accumulate in viral factories in RAW 264.7 macrophages [49] and BALB/3T3 fibroblasts] 52] infected with ECTV and in HeLa cells infected with VACV] 54]. Thus, the presence of cellular proteins within viral factories seems cell type specific. The accumulation of NF-κB within viral replication centers in ECTV-infected GM-BM is surprising, since such phenomenon has not been observed earlier [49,52,54]. Poxviruses, such as ECTV and VACV, are capable of binding to specific host proteins [14] and retain certain cellular proteins in the region of cytoplasmic DNA factories in infected cells [54]. It is not excluded that, due to the different nature (non-abortive) of ECTV replication cycle in DCs, other mechanisms may be exploited by ECTV to promote its replication in such cells.

Inhibition of IRF3 and IRF7 signaling pathways in ECTV-infected GM-BM could be due to the presence, in ECTV genome, of A46, N1, C6 and N2VACV Copenhagen (VACV-COP) counterparts, with 98%, 89%, 90% and 91% identity at the amino acid level, respectively [14].VACV A46 disrupts activation of IRF3 induced by TIR domain–containing adaptor inducing IFN-β (TRIF) [55]. N1 protein of VACV has been shown to strongly associate with TANK-binding kinase 1 (TBK1) of the multisubunit I-κB kinase complex and inhibits both, NF-ĸB and IRF3 signaling [56]. VACV protein C6 acts at the level of the TBK1/IKKε complex and inhibits IRF3 and IRF7 activation and IRF3 translocation into the nucleus [57]. Meanwhile, N2 is a nuclear protein that inhibits IRF3 downstream of the kinase TBK-1 following IRF3 phosphorylation and nuclear translocation [58]. Interestingly, in ECTV-infected GM-BM the subcellular distribution of IRF3 was diametrically changed: IRF3 mostly accumulated in yet undefined cytosolic structure/vacuole. IRF7, but not IRF3, has been demonstrated to co-localize with MyD88 in endosomal vesicles in a macrophage cell line [59]. The phenomenon of IRF3 accumulation in vacuoles has not been observed previously, and at the moment we do not know what the biological significance of this fact is.

The second possible explanation for the reduced synthesis of proinflammatory cytokines and chemokines by ECTV-infected GM-BM is global down-regulation of genes involved in TLR, RLR, NLR and IFN type I signaling observed in GM-BM, suggesting that ECTV is capable of disrupting PRR signaling pathways and inhibits subsequent triggering of antiviral innate immune response. In line with these observations, our recent study revealed that ECTV decreases expression of many PRR and chaperone genes, as well as genes of downstream signaling pathways and responsive components in peritoneal macrophages of susceptible BALB/c and resistant C57BL/6 mice. The suppression of mRNA expression is much more evident in BALB/c mice [60]. Moreover, the level of some cytokines in ECTV-infected cultures of GM-BM can also be influenced by secreted immunomodulatory proteins encoded by ECTV [61] and virus-induced apoptosis at later stages of infection.

Finally, GM-BM infected with ECTV exhibited impaired capacity to stimulate proliferation of allogeneic CD4^+^ T lymphocytes. Our results are consistent with the previous observation that other orthopoxviruses, such as CPXV, reduce the ability of DCs to stimulate T cell proliferation in MLR reaction [22]. VACV has also been shown to impair APCs in their capacity to stimulate antigen-specific CD4^+^T lymphocytes to produce IL-2 [18]. The failure of ECTV-infected GM-BM to induce proliferation of T cells may be explained by several reasons. Firstly, morphological changes that infected DCs undergo may interfere with their interaction with responder T cells. Secondly, reduction of crucial co-stimulatory molecules, such as CD80 and CD86 on DC surface may induce functional inactivation (anergy) of T cells. Moreover, ECTV-induced apoptosis of GM-BM may block downstream T cell activation events. Finally, impaired release of cytokines that, on the one hand, act as an autocrine activators of DCs, on the other hand, arecentrally important to T cell function may consequently limit T cell survival and proliferation [62]. Additionally, we cannot exclude that T cells could have become infected during co-incubation with ECTV-infected DCs, since infected DCs release progeny virions to the extracellular environment.

In summary, our results showfor the first time that ECTV infection of GM-BM, including cDCs, is productive and infected cells exhibit severe impairment across multiple functions, consequently leading to their functional paralysis. It shows that a single virus may target multiple aspects of cDC physiology, demonstrating incredible adaptation of the virus to its natural host cells. Better understanding of orthopoxvirus interactions with the host immune response may help improve orthopoxvirus vectors for use against viral infections and malignancies. Currently, vaccinia virus next to adenovirus, herpes virus and Newcastle disease virus, is being used as replicating vector in systemic cancer therapy [63]. More importantly, detailed elucidation of the orthopoxvirus interaction with cDCs should help in the development of new targets for preventative and therapeutic intervention e.g., DC vaccines in immunization against orthopoxviruses and other viruses. Eventually, the genetic similarity of ECTV to VARV and MPXV, the common features of the resulting disease, and the convenience of the mouse as a laboratory animal underscores its utility in the study of orthopoxvirus pathogenesis and in the development of therapeutics and prophylactics. This is of great importance especially when increased prevalence of MPXV in humans (particularly in immunocompromised hosts) is observed and when MPXV is able to acquire mutations increasing its transmissibility, virulence and pathogenic potential [64].

## Materials and methods

### Ethics

All experiments on animals were carried out in accordance with institutional Guidelines for Care and Use of Laboratory Animals and were approved by the 3rd Ethical Committee for Animal Experimentation at Warsaw University of Life Sciences–SGGW (permission no. 34/2012). Animal facilities at the Faculty of Veterinary Medicine (registration no. 14313537) are fully certified by the district veterinary inspector.

### Mice

Inbred, male BALB/c (H-2^d^) and C57BL/6 (H-2^b^) 8–12 weeks old mice were used for GM-BM preparation and isolation of splenic CD4^+^ T cells, respectively. All mice were purchased from the animal facility at Maria Sklodowska-Curie Memorial Cancer Centre and Institute of Oncology in Warsaw, Poland. All animals were allowed to acclimate for at least 1 week before experimental procedures. Mice were given *ad libitum* access to food and water. Animals were sacrificed by cervical dislocation, and bones were collected for GM-BM generation.

### Virus

In all experiments highly virulent Moscow strain of ECTV(ATCC 1374; American Type Culture Collection, Manassas, VA) was used. The Vero cell line (ATCC CCL-81; American Type Culture Collection) was used for virus propagation and determination of virus stock infectivity by plaque formation assay (PFU/mL). Virus stocks were stored in aliquots at -70°C until used. In some experiments uvi-ECTV was used. Complete inactivation of virus was confirmed by the absence of plaque formation in the Vero cell monolayer.

### Generation, purification and infection of GM-BM

GM-BM were generated from murine bone marrow precursors using recombinant mouse (rm) GM-CSF. Cells were obtained as previously described [65] with minor modifications. GM-BM were cultured in RPMI-1640 (HyClone, Logan, UT, USA) supplemented with 10% heat-inactivated fetal bovine serum (FBS, HyClone), 100 U/ml penicillin and 100 mg/ml streptomycin (Sigma-Aldrich, St. Louis, MO, USA), 50 μM 2-mercaptoethanol (Sigma-Aldrich) and 20 ng/ml rmGM-CSF (R&D Systems, Minneapolis, MN, USA). After 10 days of culture in 100 mm bacteriological Petri dishes (Corning [Falcon] BD, Bedford, MA, USA) GM-BM were separated using MACS CD11c^+^ labeled magnetic beads (Miltenyi Biotec, Auburn, CA, USA) [26]. After MACS separation (purity ≥ 95%) CD11c^+^ cells were exposed to: (a) complete RPMI-1640 medium (mock, negative control), (b) live-ECTV [multiplicity of infection, m.o.i. = 5], or (c) uvi-ECTV (m.o.i. = 5, before inactivation). After 60 min virus adsorption at 37°C in a humidified 5% CO_2_ atmosphere, mock-, ECTV- or uvi-ECTV-exposed cells were cultured in the absence or the presence of LPS (*Escherichia coli* 0111:B4; Sigma-Aldrich, St Louis, MO, USA) for 24 h at a final concentration of 1μg/ml, which served as a control of GM-BM maturation.

### Cultivation and infection of JAWS II cells

JAWS II cells (ATCC CRL-11904) were cultured in alpha-minimum essential medium (MEM) with ribo-and deoxyribonucleosides (Corning, Corning, NY, USA) supplemented with 4 mM L-glutamine (Sigma-Aldrich), 20% heat-inactivated FBS, 100 U/ml penicillin and 100 mg/ml streptomycin, and 5 ng/ml rmGM-CSF. JAWS II cells were infected with ECTV in the same way as GM-BM at m.o.i. = 1.

### Fluid-phase endocytosis

Fluid-phase endocytosis (pinocytosis) was analyzed using FITC-Dextran 70 (Sigma-Aldrich) [66]. GM-BM of each treatment group were suspended at a density of 1 x 10^6^ cells/ml in RPMI-1640 medium and incubated with 0.5 mg/ml FITC-dextran for 60 min at 37° or at 4°C as a negative control. After washing three times with ice-cold phosphate buffered saline (PBS, Sigma-Aldrich) containing 2% FBS, cells were analyzed by flow cytometry.

### Receptor-mediated endocytosis and antigen processing

Receptor-mediated uptake and processing of ovalbumin (OVA) were analyzed using OVA labeled with the pH-insensitive fluorescent dye BODIPY FL (DQ-OVA; Molecular Probes, Eugene, OR, USA). 1 x 10^6^ cells/ml of GM-BM in each group were incubated for 60 min at 37° or at 4°C (negative control) in RPMI-1640 medium containing 30 μg/ml DQ-OVA. Cells were washed three times with ice-cold PBS supplemented with 2% FBS and analyzed by flow cytometry. Additionally, GM-BM in each group were seeded on microscopic slides and were stained in the same way as described above. After washing cells were analyzed by fluorescence microscopy.

### Apoptosis

The percentage of early and late apoptotic cells was measured using the Annexin-V Apoptosis Detection Kit (Sigma-Aldrich) according to the manufacturer's protocol. After annexin V-FITC and propidium iodide (PI) staining GM-BM of each group were analyzed immediately by flow cytometry. Viable, early and late apoptotic cells showed Annexin V^−^/PI^−^, Annexin V^+^/PI^−^ and Annexin V^+^/PI^+^ staining patterns, respectively.

### May-Grünwald-Giemsa (MGG) staining

For cell morphology assessmentthe MGG staining method was used. Mock-, uvi-ECTV- or ECTV-infected GM-BM, untreated or treated with LPS, were cultured on microscope sides for 24 h before fixation in methanol for 5 min at room temperature. Cells were stained with MGG method and observed under the inverted microscope Olympus IX71 (Olympus, Tokyo, Japan).

### Immunofluorescence staining of transcription factors

For detection and visualization of transcription factors, GM-BM were seeded on microscopic slides placed in a 24-well plate at a density of 2 x 10^5^ cells/well. Cells were left uninfected or were treated with live-ECTV or uvi-ECTV for 60 min at 37°C, and then incubated in the absence or the presence of LPS. After 24 h, cells were fixed with 4% paraformaldehyde (PFA, Sigma-Aldrich) and permeabilized with 0.5% Triton X-100 (Sigma-Aldrich) in phosphate-buffered saline (PBS). Next, GM-BM were blocked with 3% bovine serum albumin (BSA, Sigma-Aldrich) in 0.1% Triton X-100 and incubated for 30 min with anti-NF-κB p65 clone D14E12 (Cell Signaling Technology, Inc., Beverly, MA, USA), anti-IRF3 clone EPR2418Y or polyclonal anti-IRF7 (both from Abcam, Cambridge, MA, USA) primary antibodies. After washing with 0.1% Triton X-100 in PBS, cells were incubated with anti-rabbit IgG-Rhodamine RedX (Jackson ImmunoResearch Laboratories, Inc., West Grove, PA, USA) secondary antibody diluted in blocking solution. ECTV antigens were stained for 60 min with FITC-conjugated pAbs, obtained as previously described [27]. DNA was stained with 1 μg/ml Hoechst 33342 (Calbiochem, San Diego, CA, USA) for 2.5 min. Slides were mounted in ProLong Gold Antifade Reagent (Invitrogen Life Technologies, Carlsbad, CA, USA).

### Multi-colour immunophenotyping

For immunophenotypic characterization of GM-BM, mock-, uvi-ECTV- or ECTV-infected cells were left unstimulated or were stimulated with LPS for 24h in a 24-well plate at a density of 1 x 10^6^ cells/well. After harvesting with 3 mM EDTA (Ambion*/*Thermo Fisher Scientific, Waltham, MA,USA) in PBS,GM-BM were blocked and then stained with the following monoclonal antibodies (mAbs) used in appropriate combinations: anti-CD11c-BV421 clone N418, anti-CD86-PerCP-Cy5.5 clone GL-1 (both from BioLegend, San Diego, CA, USA) and anti-H-2D[d]-PE clone 34-2-12, anti-I-A/I-E-BV711 clone M5/114.15.2, anti-CD40-APC clone 3/23, anti-CD80-APC clone 16-10A1 (all from BD Biosciences, San Jose, CA, USA). After washing with ice-cold PBS, cells were analyzed by flow cytometry. Appropriate isotype controls (purchased from BD Biosciences) were used to exclude non-specific binding of antibodies. Fluorescence Minus One (FMO) samples were included as negative controls and were prepared for each fluorochrome to define gating boundaries.

### Flow cytometry gating and acquisition strategy

In each experiment GM-BM were stained for CD11c marker. Based on size and granularity according to forward (FSC) and side (SSC) scatter profile the population of live cells was gated. Dendritic cells were gated as FSC^high^ and CD11c^+^. Data from 2 x 10^4^ events were acquired for each sample on a BD FACSCanto II and BD LSRFortessa flow cytometers (Becton Dickinson) and analyzed with FACSDiva 7.0 software.

### Enzyme-linked immunosorbent assay (ELISA)

For cytokine and chemokine profile evaluation, GM-BM were plated at a density of 2 x 10^4^ cells/well into a 24-well plate. Cells were exposed to medium only (mock), ECTV or uvi-ECTV, following treatment with or without LPS. After 12 and 24 h of incubation, cell culture supernatants were harvested and stored at -70°C until further processing. The concentration of pro-inflammatory (TNF, IL-6, IL-12p40, IL-12p70) and anti-inflammatory (IL-10) cytokines and chemokine CCL2/MCP-1 was quantified using BD OptEIA ELISA sets (BD Biosciences). Chemokines CCL3/MIP-1α and CCL5/RANTES were detected using Quantikine ELISA Kits (R&D Systems). All ELISA kits involved sandwich type assay tests and were performed according to the manufacturer’s instructions. The concentration of cytokines and chemokines was calculated by interpolation from a linear calibration curve, performed in the same assay in parallel with sample measurement. Absorbance at 450 nm was measured using Epoch Microplate Spectrophotometer (BioTek Instruments, Inc., Winooski, VT, USA).

### Real-time polymerase chain reaction (PCR)

For detection of selected genes involved in TLR, RLR, NLR and IFN type I signaling, Real-Time PCR was performed, as previously described [60] with minor modifications. Briefly, total RNA was isolated from mock- and ECTV-infected GM-BM at 24 hpi using RNeasy Mini Kit (Qiagen, Inc., Valencia, CA, USA) according to the manufacturer’s instruction. Additionally, during RNA purification genomic DNA was removed using RNase-Free DNase Set (Qiagen). RNA concentration was measured in the Take-3 system on Epoch BioTek spectrophotometer and analyzed in Gen5 software. Next, reverse transcription was done using the RT^2^First Strand Kit (Qiagen) according to the manufacturer’s protocol. For analysis of selected genes the Mouse Antiviral Response RT^2^; Profiler PCR Array (Qiagen) was used according to the manufacturer’s instructions. Briefly, 550 ng cDNA was added to RT^2^SYBR Green Mastermix and aliquoted into each well of RT^2^ Profiler PCR array plates. Amplification was conducted at 95°C for 10 min, 40 cycles of 95°C for 15 s and 60°C for 1 min, and performed in ABI 7500 thermocycler (Life Technologies, Carlsbad, CA, USA). Fluorescence data were collected at 60°C/1 min cycle. Amplification data were acquired through SDS Software (Applied Biosystems). Average threshold cycle (C_T_) values from PCR reactions were normalized against the average C_T_ values for *Atg12* from the same cDNA sample and shown as 2^(-ΔΔCT) were ΔC_T_ = C_T Gene_−C_T Atg12_.

### Mixed leukocyte reaction (MLR)

MLR assay was used to evaluate GM-BM-induced proliferation of allogeneic CD4^+^ T cells. Mock-, uvi-ECTV- or ECTV-infected GM-BM were harvested 24 h after culture with or without LPS. Allogeneic CD4^+^ T cells were obtained by negative selection from spleens of uninfected C57BL/6 mice using mouse CD4^+^ T Cell Isolation Kit (Miltenyi Biotec) with magnetic cell separation. The purity of CD4^+^ T cells was assessed by flow cytometer and was greater than 90%. Prior to co-culture with T cells, GM-BM were treated with 100 μg/ml of mitomycin C (Sigma-Aldrich) at 37°C for 30 min. Graded dilutions of GM-BM were added to 1 x10^5^ allogeneic CD4^+^ T cell in triplicate in 100 μl of complete medium to flat-bottomed 96-well plate (DCs:T cells = 1:5 and 1:10). GM-BM were co-cultured with allogeneic T cells for 5 days and then 10 μl of Cell Counting Kit-8 (CCK8) reagent (Sigma-Aldrich) was added to each well. After 2 h of incubation the conversion of WST-8 to water-soluble formazan was measured at 450 nm using Epoch Microplate Spectrophotometer. T cell proliferation in each studied group of GM-BM was calculated in relation to values obtained in mock-infected cells, considered as 100%.

### Fluorescence microscopy

GM-BM were examined using Olympus BX60 fluorescence microscope (Olympus) equipped with Color View III cooled CCD camera. Image analysis was performed using Cell^F (Olympus) and ImageJ (NIH, Bethesda, Maryland, USA) software. The brightness and contrast of images were adjusted using CellSens Dimension (Olympus) or Adobe Photoshop CS2 (Adobe Systems Inc., San Jose, CA, USA).

### Scanning electron microscopy (SEM)

GM-BM were seeded on microscopic slides placed in a 24-well plate at a density of 2 x 10^5^ cells/well. Cells were left uninfected or were treated with live-ECTV or uvi-ECTV for 60 min at 37°C, and then incubated in the absence or the presence of LPS for 24h. Next, GM-BM grown on microscopic slides were fixed for 60 min with 2.5% glutaraldehyde in phosphate buffer and postfixed for 60 min with 1% osmium tetroxide in phosphate buffer. After dehydration in ethanol and acetone series and drying in a CPD 7501 critical point drier (Polaron; Hatfield, PA, USA), GM-BM were coated with a gold layer in a JFC-1300 sputter-coater (JEOL, Tokyo, Japan). The SEM imaging was operated under FEI Quanta 200 environmental scanning electron microscope (ESEM) with EDAX EDS system (FEI, Tokyo, Japan).

### Statistical analysis

All quantitative data were expressed as the arithmetic mean ± standard deviation (SD) of at least three independent experiments (unless otherwise indicated). Prior to a t-test, variables were assessed for normal distribution using the Shapiro-Wilk W test. Depending on the normal distribution of data and homogeneity of variance the following tests were applied:two-dependent (paired) and two-independent (unpaired) Student’s *t*-tests, Wilcoxon signed-rank test and Mann-Whitney U-test (STATISTICA 6.0 software, StatSoft Inc., Tulsa, OK, USA). Significance was assessed at *P* ≤ 0.05 (*) and *P* ≤ 0.01 (**).

## Supporting information

S1 Fig Characteristics of GM-BM before and after MACS separation of CD11c^+^ cells. Representative dot plot demonstrating gating strategy of isotype controls (A) and CD11c and I-A/I-E staining (B). (C) Representative histograms demonstrating MFI of CD11c, CD11b and CD205 expression on GM-BM before and after MACS separation. Grey histograms represent staining with appropriate isotype controls.
(TIF)

S2 Fig Replication of ECTV in JAWS II cells. Representative images of mock–and ECTV–infected JAWS II cells 24 hpi stained with Hoechst 33342 (blue fluorescence) and pAbs anti-ECTV (green fluorescence). The magnified images are of the boxed regions. Arrows indicate viral particles; arrowheads show viral factories. Scale bars = 10 μm.
(TIF)
