# Supplementary figures and images for: Functional paralysis of GM-CSF–derived bone marrow cells productively infected with ectromelia virus
Source: PLoS One. 2017 Jun 12;12(6):e0179166. doi: 10.1371/journal.pone.0179166 (PMC5467855; doi:10.1371/journal.pone.0179166)

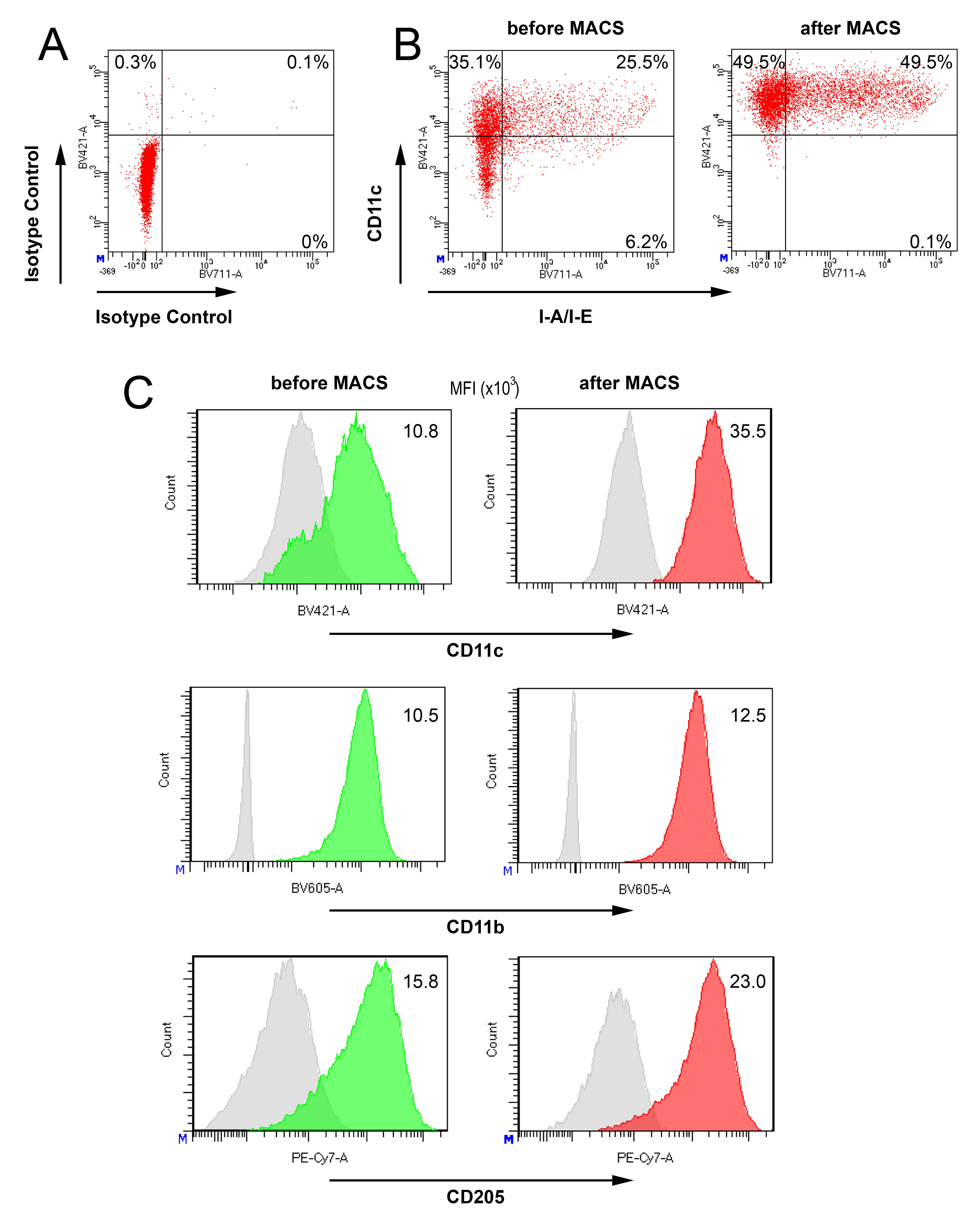

Supplement: S1 Fig — Representative dot plot demonstrating gating strategy of isotype controls (A) and CD11c and I-A/I-E staining (B). (C) Representative histograms demonstrating MFI of CD11c, CD11b and CD205 expression on GM-BM before and after MACS separation. Grey histograms represent staining with appropriate isotype controls. (TIF) [file pone.0179166.s001.tif]

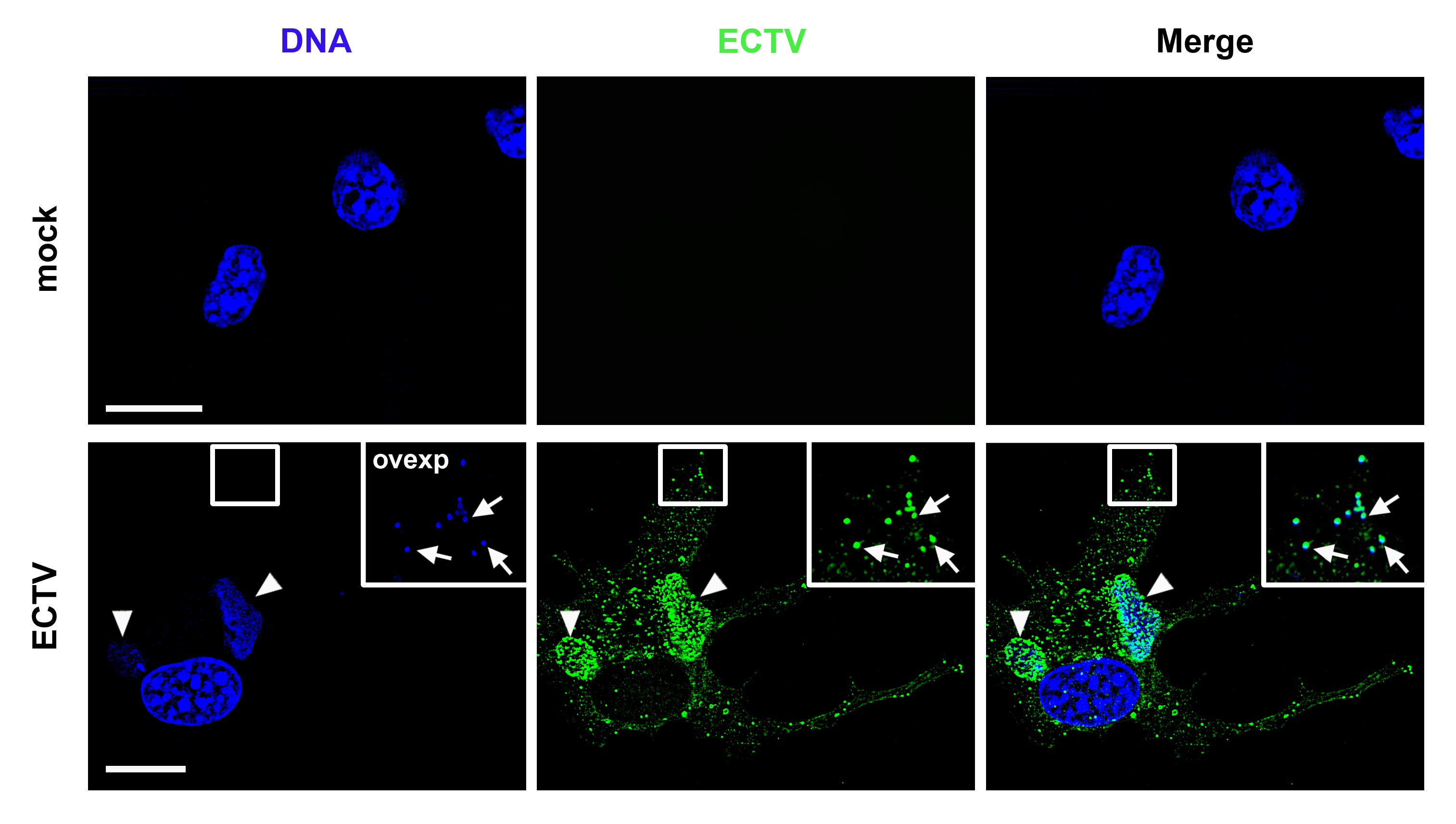

Supplement: S2 Fig — Representative images of mock–and ECTV–infected JAWS II cells 24 hpi stained with Hoechst 33342 (blue fluorescence) and pAbs anti-ECTV (green fluorescence). The magnified images are of the boxed regions. Arrows indicate viral particles; arrowheads show viral factories. Scale bars = 10 μm. (TIF) [file pone.0179166.s002.tif]
